# Association of miR-27a, miR-181a and miR-570 genetic variants with gallbladder cancer susceptibility on North indian population

**DOI:** 10.1186/1755-8166-7-S1-P17

**Published:** 2014-01-21

**Authors:** Annapurna Gupta, Kiran L Sharma, Annu Yadav, Vijay Kumar, Sanjeev Misra, Ashok Kumar, Balraj Mittal

**Affiliations:** 1 Departments of Genetics, Sanjay Gandhi Post Graduate Institute of Medical Sciences (SGPGIMS), Lucknow, India; 2 Department of Surgical Oncology, KGMU, Lucknow, India; 3 Surgical Gastroenterology, SGPGIMS, Lucknow, India

## Background

MicroRNAs are small endogenously expressed short non-coding RNAs. They appear to be critical regulators of tumor biology as their aberrant expression is well characterized in cancer progression. The role of microRNA is not fully understood in gallbladder carcinoma, so in present study we investigated the role of miR-27a, miR-181a and miR-570 genetic variants with gallbladder cancer (GBC) susceptibility.

## Material and methods

In this case-control study, we evaluated the role of miR-27a, miR-181a and miR-570 genetic polymorphisms with GBC susceptibility in North Indian population. The present study included 515 GBC patients and 200 healthy controls from North India. Genotypes were determined by TaqMan probes. Statistical analysis was done by SPSS ver. 16. In silico analysis was performed using Bioinformatics tools (F-SNP, FAST-SNP).

## Results

Logistic regression analysis showed no significant association of miR-27a, miR-181a and miR-570 genetic polymorphism with GBC susceptibility (P> 0.05). On stratifying our data on the basis of gall stone status, the [AG+GG] genotypes of miRNA rs895819 (A>G) were significantly associated with increased risk of GBC in patients without stone (p=0.003 OR=1.83 [(95%CI) 1.23-2.72]. The genetic risk by miR-27a, rs895819 (A>G) was also modulated by tobacco consumption as the heterozygotes (AG) were at higher risk p=0.005 OR=1.94 [(95%CI) 1.22-3.08]. However, there was no association of miR-181a and miR-570 polymorphisms with disease risk in subgroup analysis. In-silico analysis showed change in transcriptional regulation of miR-27a and miR-570 variations.

## Conclusions

We found significant association of miRNA rs895819 A>G with gallbladder cancer risk through gallstone independent pathway and tobacco usage.

